# Variation in *Sphingomonas* traits across habitats and phylogenetic clades

**DOI:** 10.3389/fmicb.2023.1146165

**Published:** 2023-04-17

**Authors:** Bahareh Sorouri, Cynthia I. Rodriguez, Brandon S. Gaut, Steven D. Allison

**Affiliations:** ^1^Department of Ecology and Evolutionary Biology, University of California Irvine, Irvine, CA, United States; ^2^Department of Earth System Science, University of California Irvine, Irvine, CA, United States

**Keywords:** *Sphingomonas*, pangenome, habitats, traits, phylogenetics

## Abstract

Whether microbes show habitat preferences is a fundamental question in microbial ecology. If different microbial lineages have distinct traits, those lineages may occur more frequently in habitats where their traits are advantageous. *Sphingomonas* is an ideal bacterial clade in which to investigate how habitat preference relates to traits because these bacteria inhabit diverse environments and hosts. Here we downloaded 440 publicly available *Sphingomonas* genomes, assigned them to habitats based on isolation source, and examined their phylogenetic relationships. We sought to address whether: (1) there is a relationship between *Sphingomonas* habitat and phylogeny, and (2) whether there is a phylogenetic correlation between key, genome-based traits and habitat preference. We hypothesized that *Sphingomonas* strains from similar habitats would cluster together in phylogenetic clades, and key traits that improve fitness in specific environments should correlate with habitat. Genome-based traits were categorized into the Y-A-S trait-based framework for high growth yield, resource acquisition, and stress tolerance. We selected 252 high quality genomes and constructed a phylogenetic tree with 12 well-defined clades based on an alignment of 404 core genes. *Sphingomonas* strains from the same habitat clustered together within the same clades, and strains within clades shared similar clusters of accessory genes. Additionally, key genome-based trait frequencies varied across habitats. We conclude that *Sphingomonas* gene content reflects habitat preference. This knowledge of how environment and host relate to phylogeny may also help with future functional predictions about *Sphingomonas* and facilitate applications in bioremediation.

## 1. Introduction

Bacteria occur in a wide diversity of habitats, but the factors that control habitat preference are unclear (Fierer and Jackson, 2006; Martiny et al., 2006; Merino et al., 2019). Given that habitats vary in their abiotic and biotic conditions, different habitats may select for different organismal traits (Noble and Slatyer, 1977). These traits can be phylogenetically conserved (Martiny et al., 2013; Dolan et al., 2017; Isobe et al., 2019, 2020), horizontally transferred (Ochman et al., 2000), and reflect trade-offs underlying life-history strategies. For environmental microbes, one way to organize these trade-offs is the Y-A-S framework, which posits that bacterial life-history strategies are driven by tradeoffs in resource allocation to growth Yield, resource Acquisition, and Stress tolerance responses (Malik et al., 2020). Investigating functional traits related to the Y-A-S strategies has the potential to yield insights into factors that affect the distributions of microbial taxa.

*Sphingomonas* is an excellent bacterial genus to investigate the distribution of habitat preference traits because it is found in a wide range of habitats. Within the Proteobacteria phylum, the *Sphingomonas* genus contains gram-negative, strictly aerobic, chemoheterotrophic, yellow-pigmented bacteria that possess glycosphingolipids in their cell envelope (Yabuuchi et al., 1990; Balkwill et al., 2006). *Sphingomonas* species have been isolated from soils, plant roots, water distribution systems, human samples, and hospital machines (White et al., 1996; Leys et al., 2004). Some species cause animal disease, while others are antagonistic toward phytopathogenic fungi that infect commercially important plants (White et al., 1996). Additionally, *Sphingomonas* species have also been used on the International Space Station to aid the extraction of rare earth elements (Cockell et al., 2020). On planet Earth, *Sphingomonas* serves as biocatalyst for bioremediation and can be found in soils that are contaminated with pollutants (Leys et al., 2004). Understanding the distribution of *Sphingomonas* is especially important because with appropriate management strategies, this lineage can be a tool to clean up polluted environments (Onder Erguven and Demirci, 2019). Furthermore, *Sphingomonas* is able to degrade cellulose and hemicellulose and is therefore involved in organic carbon decomposition (Koskinen et al., 2000). Hence, the distribution and functional abilities of *Sphingomonas* make it an ideal genus for investigating phylogenetic histories of habitat preference traits.

Despite the potential importance and widespread distribution of *Sphingomonas* species, there has not yet been a comprehensive, in-depth study of the comparative genomics and phylogenetics of the genus from a trait-based perspective. Most studies thus far look at the distribution and phylogeny of select genomes from 16S rRNA perspective, and often do not consider genome-based traits (Leung et al., 1999; Leys et al., 2004; Asaf et al., 2020). Moreover, the *Sphingomonas* genus classification is still evolving; *Sphingomonas* has five sub-genus classifications, and although additional strains continue to be identified, it is difficult to place them into specific clades (Takeuchi et al., 2001; Jogler et al., 2013; Asaf et al., 2020). Additionally, some *Sphingomonas* species have been shown to improve plant growth during stressful drought and salinity conditions (Halo et al., 2015; Asaf et al., 2017). Currently, there are knowledge gaps in the literature with respect to *Sphingomonas* phylogenetics, taxonomy, and genome mapping in the context of stress tolerance and bioremediation (Asaf et al., 2020). Therefore, it is useful to explore the phylogenomics of *Sphingomonas* from a whole-genome and trait-based perspective. Since *Sphingomonas* has important bioremediation qualities, understanding the genetics and distributions of these traits can provide preliminary knowledge toward harnessing *Sphingomonas* to rehabilitate natural habitats (Schmidt et al., 1992).

The knowledge of how environment and host correspond to traits may also help with future functional predictions. In this study, we downloaded over 400 available *Sphingomonas* sequences from public databases, assigned them to a habitat based on where they were isolated, and assessed their phylogenetic relationships. With this information, we sought to address two questions. First, are there significant relationships between habitat and phylogeny? Second, do key, genome-based traits demonstrate phylogenetic clustering and correlate with habitat preference?

**FIGURE 1:**
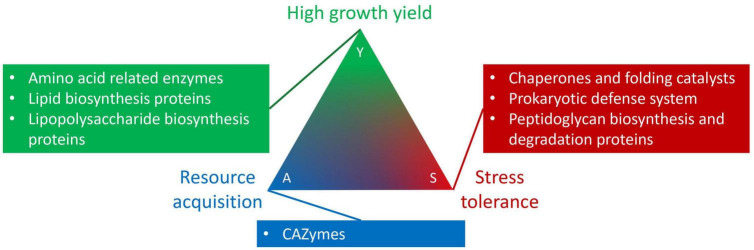
Genome-based trait groupings into the Y-A-S life history strategy framework developed by Malik et al. (2020).

We used the genome-based traits as proxies for the Y-A-S life history categories (Figure 1; Malik et al., 2020). For growth yield, we investigated the distribution of genes underlying amino acid related enzymes, lipid biosynthesis proteins, and lipopolysaccharide biosynthesis proteins. Genes for carbohydrate-active enzymes (CAZymes) reflected resource acquisition strategies. Finally, for stress tolerance we explored genes associated with chaperones, folding catalysts, the prokaryotic defense system, as well as peptidoglycan biosynthesis and degradation proteins. Collectively, these traits underlie habitat preference. We hypothesize that *Sphingomonas* strains from similar habitats will cluster together in phylogenetic clades. Furthermore, key traits that improve fitness in specific environments should correlate with the isolation habitat. For example, CAZyme genes should be most prevalent in genomes of *Sphingomonas* associated with plants, and prokaryotic defense system genes would be the highest in *Sphingomonas* genomes found at locations with a contaminant. Ultimately, our findings will improve the understanding of *Sphingomonas* distribution across habitats, as well as illuminate the link between habitat preference and life history strategies.

## 2. Materials and methods

### 2.1. Library collection and curation

We downloaded 440 publicly available *Sphingomonas* genomes from the PATRIC database on 31 July 2020 (Wattam et al., 2014) and used the metadata for each strain to identify the isolation source (Table 1). The sequences were categorized by their isolation source and assigned to one of eight groups based on the strain description: animal (*n* = 10), clinical (*n* = 43), contaminated site (*n* = 13), industrial (*n* = 13), environmental (*n* = 54), plant (*n* = 68), water (*n* = 34), and other (*n* = 17; Table 1). More specifically, strains in the animal category were isolated from living, non-human sources. Strains in the clinical category came from hospital settings and included bodily samples from human beings, like blood. Any strain with the word “contaminated” in the description was placed in the contaminated site category. The environment category consisted of strains from abiotic, outdoor sources that were not water-based, like soils. The industrial category included samples from bioreactors, mines, and wastewater facilities (which contained the key phrase “activated sludge” in the description). Strains isolated from hosts in the plant kingdom were placed in the plant category; these strains were isolated from different plant parts such as the seed, root, stem, and leaf. The water category consisted of strains isolated from a water source and sediments that did not include “contaminated” in the description. Finally, strains that could not be assigned to one of the previous 7 distinct groups were placed in the other category, such as samples from lichens and dust (Table 1). Genomes with unspecified isolation sources were removed from our analyses.

**TABLE 1:** Classification descriptions of the isolation sources for *Sphingomonas* samples.

Classification	Description
Animal	Isolation source is from a living, non-human, non-plant source
Clinical	Samples from a hospital that caters toward human beings, includes blood samples
Contaminated site	Any sample that contains “contaminated” in the description
Environment	Isolated from abiotic, outdoor sources, such as soil
Industrial	Sources from bioreactors, mines, and wastewater facilities (contains “activated sludge”)
Plant	Samples isolated from the plant kingdom, can come from seeds, leaves, and roots
Water	Any sample from a water source and sediment that does not contain “contaminated” in the description
Other	Samples that do not fit in to the other categories, can be lichens, dust

Next, we checked the completeness of the genomes against the *Sphingomonadales* order using the BUSCO (Benchmarking Universal Single Copy Orthologs) v4.1.4 program (Seppey et al., 2019). Genomes with a BUSCO completeness score of less than 95% were filtered out. We used the online QUAST (Quality Assessment Tool for Genome Assemblies) server v5.0.2 to investigate the quality of the remaining genomes (Gurevich et al., 2013). We also ran CheckM v1.2.2 against the *Sphingomonadales* and *Alphaproteobacteria* lineages to confirm the completeness of the remaining genomes and check for contamination (Parks et al., 2015).

From the initial genome library, 254 high quality *Sphingomonas* genome sequences remained for further analysis. These genomes consisted of 23 complete genomes and 231 fragmented genomes. All genomes were annotated with Prokka v1.14.6 default parameters and the *Sphingomonas* genus tag (Seemann, 2014). Core and accessory genes were identified with Roary v3.13.0 using a 50% blastp sequence identity, the default core gene identity of 99%, and a maximum gene cluster of 25,000,000 (Page et al., 2015). For comparison to the larger subset that included fragmented genomes, we also used Prokka and Roary to quantify the pangenome for just the 23 complete genomes (Seemann, 2014; Page et al., 2015).

### 2.2. Outgroup optimization

*Zymomonas*, *Rhizobium*, and *Rhodospirillum* are three closely related genera to *Sphingomonas* (Leys et al., 2004). To select the best outgroup or combinations of outgroups, we used Roary core gene counts. Specifically, we compared the core genes of the *Sphingomonas*-only ingroup to the core genes of the ingroup with various combinations of outgroups. We also included *Escherichia coli* as a distantly related outgroup for further confirmation (Zhao et al., 2017). We selected *Rhodospirillum centenum SW* (GenBank Accession: CP000613) as an outgroup because it yielded a core gene count that was closest to the *Sphingomonas*-only ingroup. Furthermore, previous phylogenetic analysis (Leys et al., 2004) confirmed that *Rhodospirillum* is not part of the ingroup.

### 2.3. Reference tree visualization

We made a phylogenetic tree with core genes present in *Sphingomonas* genomes and the *Rhodospirillum* outgroup using methods from Rodriguez and Martiny (2020). In short, we ran Roary again with the same previously mentioned parameters for the *Sphingomonas* ingroup and *Rhodospirillum* outgroup. We identified 401 core genes and generated a bootstrapped maximum likelihood tree of the alignment with RAxML v8.2.12 with the PROTGAMMABLOSUM62 substitution model and 100 rapid bootstrap searches (Stamatakis, 2014). Two of the 254 *Sphingomonas* sequences were removed from the analyses since RAxML deemed them identical. Therefore, to minimize bias, we removed the duplicate sequences and re-ran Roary with the 252 *Sphingomonas* genomes to generate an alignment of 404 core genes (Supplementary material). We used the core gene alignment to construct a phylogenetic tree with RAxML and subsequently visualized the tree with the iTOL v6.5 interactive tool (Figure 3; Letunic and Bork, 2019).

### 2.4. Clade designation

We manually designated phylogenetic clades based on their divergence from the common ancestor. We marked the first clade by starting from the most distant, large monophyletic group. Subsequently, we moved along the tree until we came across another large, monophyletic group that was interpreted as another clade. Clades were defined in this manner until we identified a total of 12. Two strains that resembled an outgroup within two separate monophyletic clades were not included as part of the clade. We confirmed the clades and genome clusters by identifying pairwise average amino acid and nucleotide identities with the Enveomics tool (Rodriguez-R and Konstantinidis, 2016). Additionally, clades possessed a bootstrap identity of at least 86.

### 2.5. Genome-based traits

We quantified the abundances of genome-based traits involved in high growth yield, resource acquisition, and stress tolerance strategies. To identify the traits, we used the CAZy (Cantarel et al., 2009) and KEGG databases (Kanehisa and Goto, 2000). For CAZymes we determined glycoside hydrolase and carbohydrate binding module abundances. Specifically we identified cellulase and glycoside hydrolase genes from Prodigal protein annotations using dbCAN2, a metaserver based on the CAZy database (Hyatt et al., 2010; Zhang et al., 2018). In our analysis, we only selected genes that were found with all three tools available on dbCAN2: HMMER, DIAMOND, and Hotpep. Additionally, we used the GhostKOALA v2.2 automatic annotation server to annotate the remaining genes based on KEGG Orthology (Kanehisa et al., 2016). We selected these genome-based traits for further analyses: lipopolysaccharide biosynthesis proteins (*n* = 66), lipid biosynthesis proteins (*n* = 29), amino acid related enzymes (*n* = 52), prokaryotic defense system (*n* = 77), peptidoglycan biosynthesis and degradation proteins (*n* = 34), and finally chaperones and folding catalysts (*n* = 42). These genes were grouped into the Y-A-S microbial life history trait-based framework developed by Malik et al. (2020) based on their role in growth yield, resource acquisition, and stress tolerance strategies (Supplementary material). We calculated the average relative abundance of each trait for each clade and visualized them with the ggpubr v0.4.0 R package (Kassambara, 2020).

### 2.6. Statistical analyses

After quantifying gene abundances, we natural log transformed the gene counts of the genome-based traits. Subsequently, we confirmed the normality of residuals using histograms and the Shapiro–Wilk tests, then ran Kruskal–Wallis rank sum tests to identify differences across habitats. We performed Kruskal–Wallis tests since not all the functional gene data were normally distributed. Additionally, we conducted phylogenetic generalized least squares (PGLS) statistical analyses to test whether there was an association between the habitat and the genome-based traits, independent of phylogenetic history (Mundry, 2014). We also used PGLS statistics to limit statistical bias by confirming if significant Kruskal–Wallis results were influenced by phylogenetic relatedness.

We used R v4.1.0 to run all the statistical analyses, and specially incorporated the “nlme,” “geiger,” “phytools,” and “ape” packages (Revell, 2012; Pennell et al., 2014; Paradis and Schliep, 2019; Pinheiro et al., 2021). We also used the *cor.test* function of the base R “stats” package to calculate the Pearson’s product-moment correlation to determine whether genome size and gene counts were correlated (R Core Team, 2021).

Additionally, we ran ANOSIM tests to determine whether phylogeny was related to habitat preference. Using the “ape” package in R, we called the tree in R and subsequently used the “cophenetic” function in the “stats” package to calculate a distance matrix (Paradis and Schliep, 2019; R Core Team, 2021). Then, we used the “anosim” function in the R package “vegan” to run ANOSIM tests (Oksanen et al., 2020).

## 3. Results

### 3.1. Pangenome

We downloaded 440 publicly available *Sphingomonas* genomes, selected 252 high-quality genomes, and carefully curated them into 8 habitat categories based on the isolation source. The minimum N50 value was 17,936 bases and the maximum was 6,205,897 bases. The minimum GC percent content was 61.98% and the maximum was 70.01%. We selected genomes with a BUSCO completeness score of at least 95%. Most of the sequences had a CheckM completeness score of at least 99% (*n* = 220) and only 1 genome had a completeness score under 96.5% with the lowest score of 94.2%. Additionally, CheckM contamination scores revealed a mode of 0.0, an average score of 1.35, and a maximum score of 13.27 (Supplementary material).

Roary and Prokka pangenome analysis for the 252 *Sphingomonas* genomes revealed a total of 113,816 genes. Specifically, there were 444 core genes found in at least 99% of the genomes, 304 soft core genes found in 95 to 99% of genomes, 4,070 shell genes found in 15 to 95% of genomes, and 108,998 cloud genes present in less than 15% of genomes (Table 2).

**TABLE 2:** Pangenome analysis for the 252 *Sphingomonas* genomes.

Gene	Description	Frequency
Core genes	99% ≤ strains ≤ 100%	444
Soft core genes	95% ≤ strains ≤ 99%	304
Shell genes	15% ≤ strains ≤ 95%	4,070
Cloud genes	0% ≤ strains ≤ 15%	108,998
Total	0% ≤ strains ≤ 100%	113,186

**FIGURE 2:**
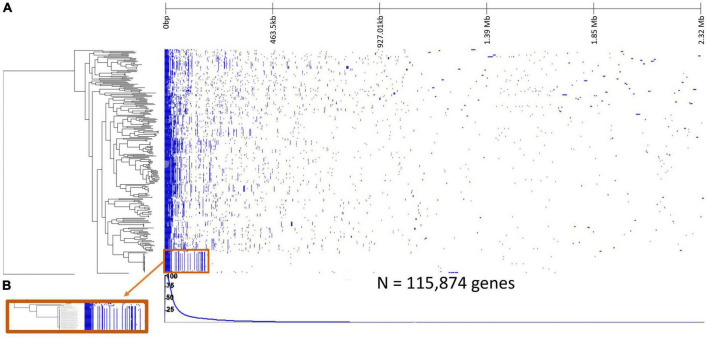
Pangenome analysis of 252 *Sphingomonas* genomes and the *Rhodospirillum centenum SW* outgroup. **(A)** Gene presence-absence heatmap where vertical blue lines represent presence of a gene within rows corresponding to the *Sphingomonas* genome, and white reflects gene absence. The line graph underneath indicates the percentage of strains possessing the corresponding gene. **(B)** Close-up of the gene patterns within a clade shows how clades contain similar gene clusters.

When the *Rhodospirillum centenum SW* outgroup was included in the pangenome analysis, there was a total of 115,874 genes with 404 core genes, 321 soft core genes, 4,091 shell genes, and 111,058 cloud genes (Supplementary Table 1; Figure 2). Some of the core gene functions include but are not limited to those associated with ribosomes, transcription factors, translation factors, and ATP synthases.

**FIGURE 3:**
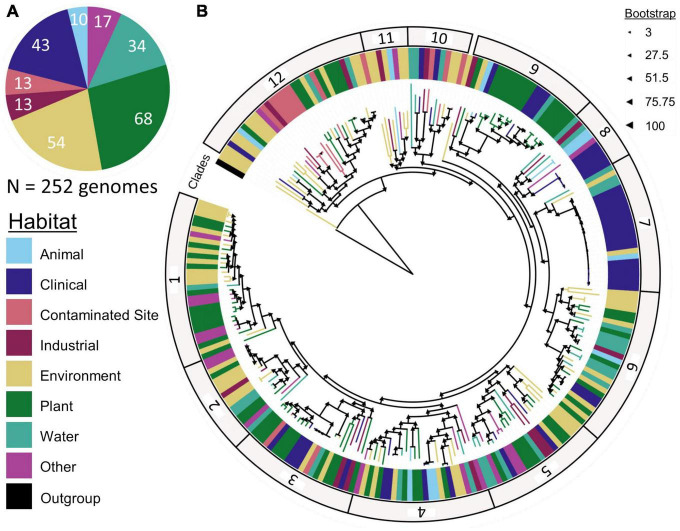
*Sphingomonas* **(A)** habitat and **(B)** phylogenetic tree constructed with 252 *Sphingomonas* genomes and 404 core genes, separated into 12 clades. The closely related *Rhodospirillum centenum SW* was used as the outgroup to identify the core gene alignment and construct the tree. Significant (*p* < 0.05) ANOSIM results indicate that *Sphingomonas* habitat preferences vary across clades.

The pangenome analysis of just the 23 complete *Sphingomonas* genomes revealed a total of 33,131 genes comprised of 758 core genes, 184 soft core genes, 4,452 shell genes, and 27,737 cloud genes (Supplementary Table 2).

### 3.2. Phylogenetic tree

Phylogenetic analysis of 252 *Sphingomonas* sequences with a *R. centenum SW* outgroup yielded a phylogenetic tree assembled from an alignment of 404 core genes (Figure 3). The tree leaves clustered into 12 clades with a minimum bootstrap value of 86. After running Enveomics, pairwise comparisons within clades revealed a minimum average amino acid identity of 33.24% and a minimum average nucleotide identity of 76.37%.

Significant ANOSIM tests (*p* < 0.05) showed that *Sphingomonas* strains from the same habitat clustered together based on phylogeny, meaning that taxa within a clade had similar habitat preferences. For example, clade 7 was mostly composed of clinical samples that were highly similar to each other, although it also contained representatives isolated from other habitats, such as water and the environment. Clade 12 was dominated by strains from contaminated regions (Figure 3; Supplementary Figure 1). Some known lineages clustered in specific clades. Clade 2 contained *Sphingomonas melonis*, which is a pathogen of yellow Spanish melon fruits and causes brown spots (Buonaurio et al., 2002). Clade 3 included *Sphingomonas sanguinis*, which causes dry rot of mango (Liu et al., 2018). *Sphingomonas naasensis* was found in clade 6 and was first isolated from forest soil in South Korea (Kim et al., 2014). Clade 7 contained *Sphingomonas koreensis*, which was first isolated from natural mineral water and can be a human pathogen in patients with meningitis (Lee et al., 2001; Marbjerg et al., 2015). Clade 8 included strains of *Sphingomonas japonica* (Supplementary Figure 2) that were isolated from the red king crab from the Sea of Japan (Romanenko et al., 2009). Moreover, *Sphingomonas* strains within the same clade shared similar clusters of accessory genes (Figure 2B).

### 3.3. Functional genes

**FIGURE 4:**
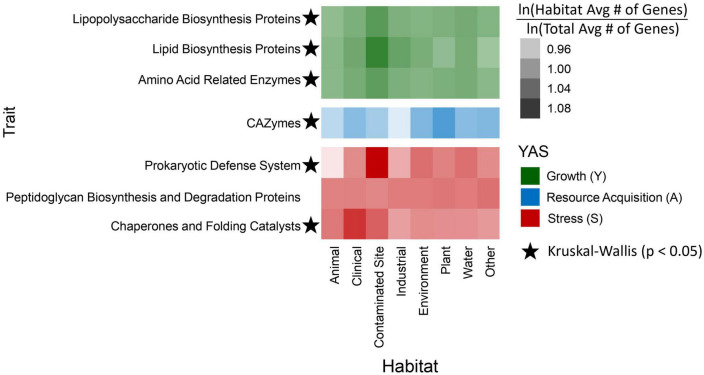
Heatmap depicting the enrichment of genome-based traits by habitat. Each heatmap box was calculated by taking the natural log of the average number of genes within a habitat for a specific trait and dividing it by the natural log of the total gene average in all habitats for the same trait. Traits are grouped together based on their Y-A-S classification: top green rows are growth traits, the middle blue CAZymes row is a resource acquisition trait, and the bottom, red rows are stress tolerance traits. Traits with stars indicate significant (Kruskal–Wallis, *p* < 0.05) differences of natural log transformed gene counts between habitats.

We identified 3,615 unique genes from the KEGG database and 269 CAZymes. A subset of KEGG Orthology genes were chosen to investigate genome-based habitat preference traits based on their classification within the Y-A-S framework, specifically for growth (*n* = 147) and stress tolerance (*n* = 153). Genes within the growth category consisted of many tRNA synthetases, and several genes in the stress tolerance category were involved with antitoxins, CRISPR, and binding to antibiotics such as penicillin (Supplementary material). Kruskal–Wallis rank sum tests on habitat and genome-based trait counts yielded significant (*p* < 0.05) differences for all traits except peptidoglycan biosynthesis and degradation proteins (Figure 4). Similarly, analysis of variance tests (ANOVA) on the phylogenetic generalized least squares (PGLS) models indicated that trait frequencies differed significantly (*p* < 0.05) by habitat for all traits except the prokaryotic defense system.

As expected, there was a significant correlation (*p* < 0.05) between genome size and gene counts for most of the traits we analyzed (Supplementary Figure 3). The largest genome with 6,899,075 bases belonged to a strain isolated from a contaminated site, and the shortest genome came from the animal classification with 2,861,323 bases. On average, genomes from contaminated sites were the largest and those from animals were the smallest (Supplementary Table 3). Therefore, genomes from contaminated sites typically had a higher enrichment of genome-based traits, whereas strains from animals often had the lowest gene enrichment when compared to the other habitats (Figure 4). The prokaryotic defense system gene group was highest within contaminated habitats. Additionally, as we anticipated, CAZyme gene frequencies were highest in strains from plants. There was also a high enrichment of chaperones and folding catalysts within genomes isolated from the clinical habitat; on average, the genome size of clinical strains was the second largest (Figure 4; Supplementary Table 3).

We also calculated the relative abundances of the habitat preference traits for each clade (Supplementary Figure 4). For the genome-based traits associated with high growth yield, the amino acid related enzymes and lipopolysaccharide biosynthesis proteins were the most abundant in clade 11, whereas lipid biosynthesis proteins were most abundant in clade 12. CAZymes linked to the resource acquisition strategy were abundant overall, with clades 1 and 3 having the highest relative abundances compared to other clades. With respect to the genome-based stress tolerance traits, chaperones and folding catalysts were most abundant in clade 7 and clade 11 had the highest abundance of genes for the prokaryotic defense system, as well as peptidoglycan and biosynthesis proteins (Supplementary Figure 4).

## 4. Discussion

Using comparative genomics, we investigated the association between *Sphingomonas* habitat and phylogeny. Our hypothesis that *Sphingomonas* strains from similar habitats would cluster together in phylogenetic clades was supported as depicted in the phylogenetic tree with a significant association between habitat and phylogeny (Figure 3, ANOSIM test *p* < 0.05). Furthermore, within clades, strains shared similar accessory genes (Figure 2). Moreover, we found partial support for the hypothesis that key, genome-based traits related to fitness in specific environments would correlate with the isolation habitat (Figure 3). A closer investigation of functional genes associated with life history strategies revealed significant differences in gene counts across habitats (Figure 3). Some of the patterns reflected what we anticipated, while others did not. Ultimately, these findings bring us one step closer toward understanding the relationship between habitat preference and phylogeny.

The phylogenetic tree indicates that there is an association between *Sphingomonas* habitat and phylogeny, supporting our hypothesis that strains from similar habitats are more closely related (Figure 3). These findings are also supported in other bacterial systems such as *Bifidobacteria*, *Curtobacterium*, and *Xylella fastidiosa* (Chase et al., 2018; Rodriguez and Martiny, 2020; Batarseh et al., 2022). It appears that abiotic factors as well as biological conditions, such as hosts, contribute to the environmental filtering and evolution of *Sphingomonas* within each habitat (Martiny et al., 2006; Kraft et al., 2015).

Although there was a significant association, the match between habitat and phylogeny was not a perfect. It is possible that our phylogeny could be improved by incorporating accessory genes. However, previous research suggests that the phylogeny produced from an alignment of accessory genes did not substantially differ from a phylogeny constructed with core genes (Batarseh et al., 2022; Scales et al., 2022). It is also possible that our 8 habitat categories (Table 1) may be too broad or too narrow, or perhaps dispersal between sources influences the evolutionary history (Finlay, 2002). Most of the environmental samples consisted of soils, while the plant samples could be separated into root, stem, and leaf subcategories (Supplementary Figure 5). The rhizosphere consists of soils in the vicinity of plant roots, and could include lineages that are selected by both soil and plant properties (Berendsen et al., 2012). Additionally, dispersal between habitats could bring together *Sphingomonas* strains from different sources in the same location (Finlay, 2002; Albright et al., 2019; Walters et al., 2022). Dispersal is particularly likely across environment, plant, water, and contaminated site habitats. Finally, we note that we eliminated some viable genomes from our analysis because we could not determine their isolation source. Future studies like ours would benefit from a standardized approach to metadata reporting about isolation methods in microbial genomics databases.

Since we found that habitat preference is phylogenetically conserved, we sought to disentangle potential genome-based traits that underlie habitat preference. *Sphingomonas* clades share similar accessory genes (Figure 2), and genome-based trait counts varied by habitat, together suggesting that adaptation to the local environment has shaped habitat preference (Figure 4). Strains from contaminated sites had more genes associated with the prokaryotic defense system, while clinical strains had higher averages for chaperones and folding catalysts (Figure 4). It is possible that in the Y-A-S life history framework, strains from both of these habitats may depend on stress tolerance strategies for survival (Figure 1; Malik et al., 2020). Chaperones and folding catalysts serve as signaling molecules to blood cells to promote immunity and inflammation (Henderson and Pockley, 2010), two common processes in clinical settings. Immune responses are stressful to bacterial infectious agents, and bacterial stress proteins such as chaperones may even trigger the immune response of hosts (Henderson et al., 2006). Moreover, compared to the other habitats, contaminated sites also had more genome-based traits associated with high growth yield (Figure 4). Since *Sphingomonas* can break down pollutants (Schmidt et al., 1992), it is possible that strains in contaminated habitats invested in resource use efficiency rather than stress tolerance. Additionally, we found that *Sphingomonas* strains isolated from plant habitats had more CAZymes (Figure 4), which suggests that they use the resource acquisition strategy to breakdown complex carbohydrates found in plant material (Hervé et al., 2010).

For traits that did not differ significantly across habitats, such as peptidoglycan biosynthesis and degradation proteins, there are two potential possibilities (Figure 4). These traits may be part of the core genome and are required by all strains for basic functioning. Alternatively, there may be finer-scale differences in specific genes that are not detected because our traits are defined as broad sums of multiple genes. Moreover, proteins may have overlapping functions in metabolic pathways, making it difficult to assign them to a single life history strategy.

Although the genomics field and sequencing technologies have made tremendous advancement (Heather and Chain, 2016), there are still challenges with assembling complete genomes. In publicly available data, there will be differences in the quality of the genomes since sampling and sequencing methods vary across studies. Therefore, to mitigate variability, we were very selective with the *Sphingomonas* genomes that we decided to investigate further. Even though we included fragmented genomes, all sequences had a minimum BUSCO score of 95% from the *Sphingomonadales* order (Seppey et al., 2019). Still, fragmented genomes may reduce the total core gene count in *Sphingomonas* pangenome analysis due to missing genes. Therefore, for comparison, we performed pangenome analysis on the 23 complete *Sphingomonas* genomes in our dataset (Supplementary Table 2), revealing 758 core genes. This analysis indicates that our core gene count of 404 for the genus is reasonable. As the diversity and frequency of genomes increases, the number of core genes should decrease.

We investigated the genomic variation and phylogeny of *Sphingomonas* across different habitats. Additionally, we used a trait-based framework to explore differences in genome-based traits and life history strategies. We found that strains from similar habitats group together in clades and share accessory genes. Although our results did not reveal distinct life history strategies for all habitats, genome-based trait counts varied by habitat. These findings indicate that *Sphingomonas* genome content reflects habitat preference. Considering the relationships between habitat, genomics, and phylogeny may help us predict *Sphingomonas* habitat preference and better exploit its potential for bioremediation.

## Data availability statement

The original contributions presented in this study are included in the article/Supplementary material, further inquiries can be directed to the corresponding author.

## Author contributions

BS performed data analysis and wrote the manuscript. BS, BG, and SA contributed to the data interpretation. All authors developed and designed the study, contributed to the article, and approved the submitted version.

## Supplementary material

Supplementary Table 1 *Sphingomonas* genomes isolation source and habitat classification descriptions (see Table 1.XLSX).

Supplementary Table 2 CheckM *Sphingomonas* genome output table (see Table 2.XLSX).

Supplementary Data Sheet 2 Roary gene presence and absence output file for *Sphingomonas* genomes and the *Rhodospirillum centenum SW* outgroup.

Supplementary Data Sheet 3 *Sphingomonas* clades, habitat classification, genome length, Y-A-S trait frequencies, and natural log trait frequencies.

Supplementary Data Sheet 4 GhostKoala gene descriptions and Y-A-S classifications.
