# Supplementary material for: Variation in Sphingomonas traits across habitats and phylogenetic clades
Source: Front Microbiol. 2023 Apr 17;14:1146165. doi: 10.3389/fmicb.2023.1146165 (PMC10150699; doi:10.3389/fmicb.2023.1146165)
Supplement: Supplementary file 1 [file Data_Sheet_1.PDF]

Supplemental Figures and Tables

Supplemental Table 1: Pangenome analysis for the 252 *Sphingomonas* strains and the *Rhodospirillum centum* outgroup.

Gene	Description	Frequency
Core genes	99% <= strains <= 100%	404
Soft core genes	95% <= strains <= 99%	321
Shell genes	15% <= strains <= 95%	4,091
Cloud genes	0% <= strains <= 15%	111,058
Total	0% <= strains <= 100%	115,874

Supplemental Table 2: Pangenome analysis for the 23 complete genomes that we included in our data analysis.

Gene	Description	Frequency
Core genes	99% <= strains <= 100%	758
Soft core genes	95% <= strains <= 99%	184
Shell genes	15% <= strains <= 95%	4,452
Cloud genes	0% <= strains <= 15%	27,737
Total	0% <= strains <= 100%	33,131

Supplemental Table 3: Average genome length for strains in the habitat categories.

Habitat	Average Genome Size (bases)
Animal	3,678,369
Clinical	4,414,984
Contaminated Site	4,947,991
Environment	4,059,478
Industrial	4,188,510
Plant	4,251,944
Water	4,296,399
Other	4,092,009

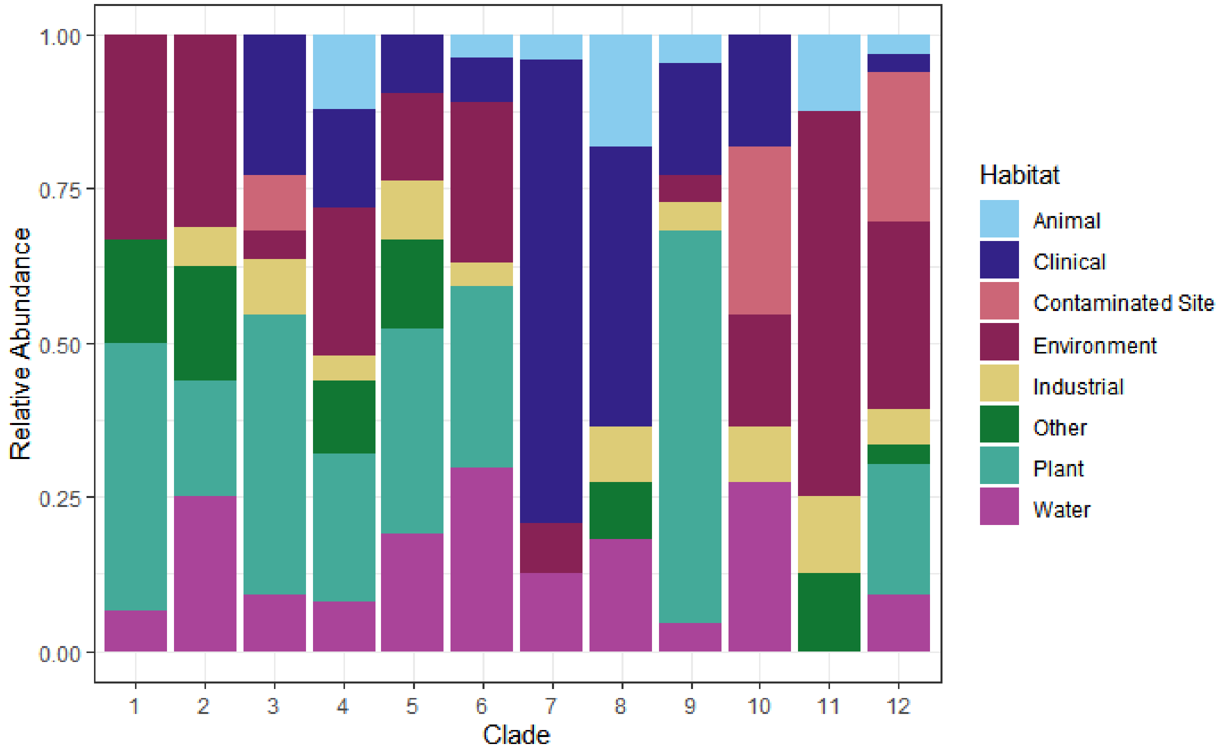

Supplemental Figure 1. Relative abundances of habitats within each clade.

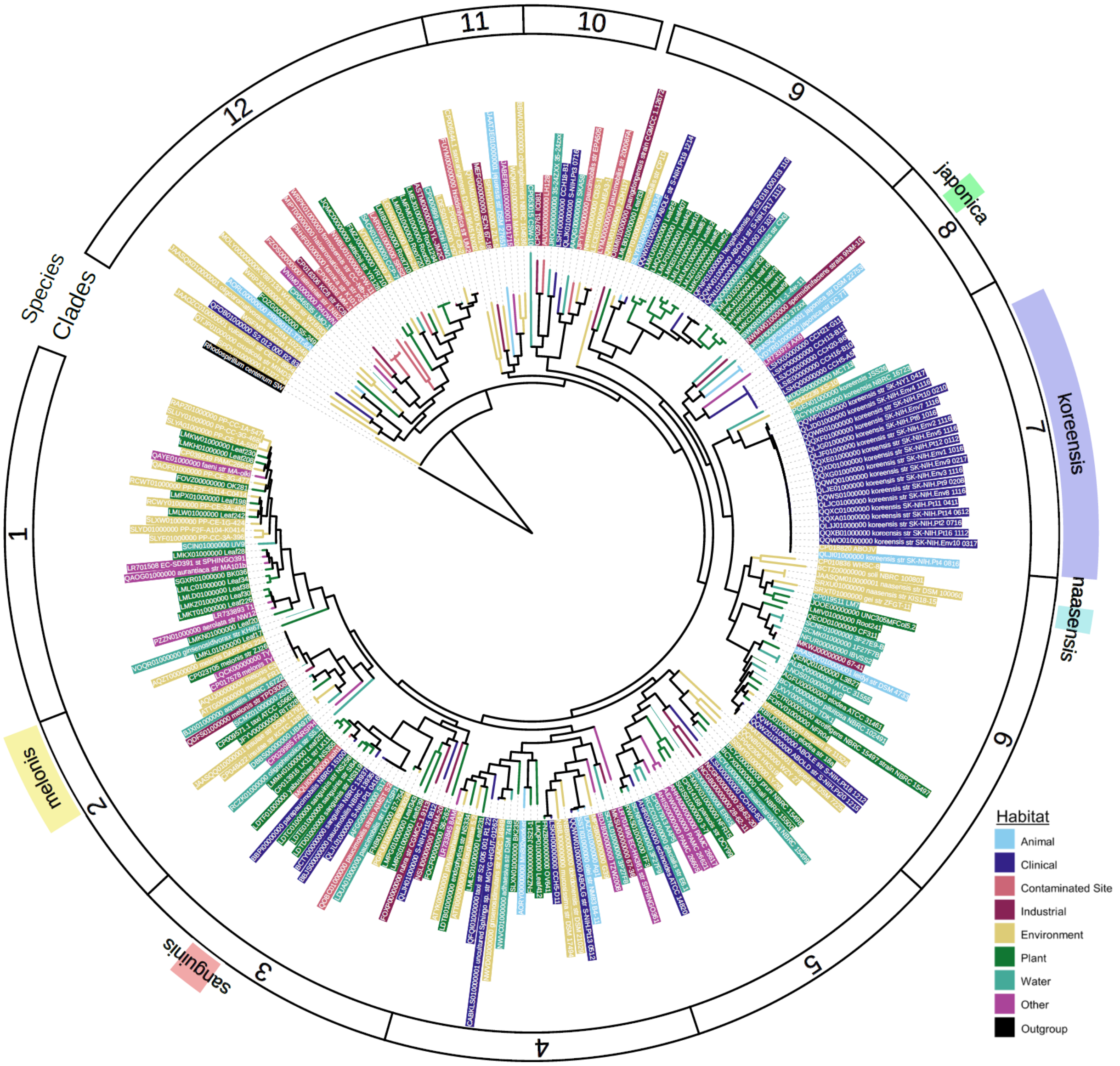

Supplemental Figure 2. *Sphingomonas* phylogenetic tree from Figure 2.3 in main text with visible names. Genomes are labeled by the GenBank Accession Number and the associated *Sphingomonas* strain.

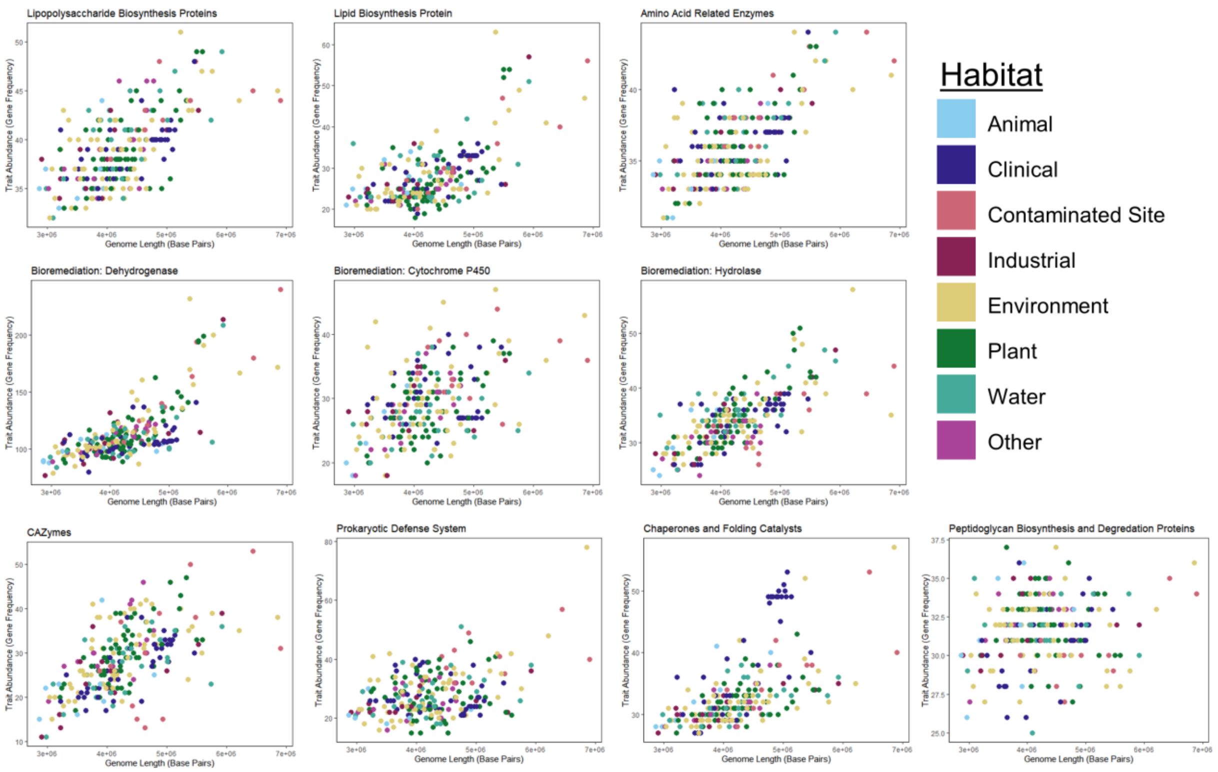

Supplemental Figure 3. Genome frequencies and traits. All traits had a significant ($p < 0.05$) correlation between the frequency of the associated genes and genome size.

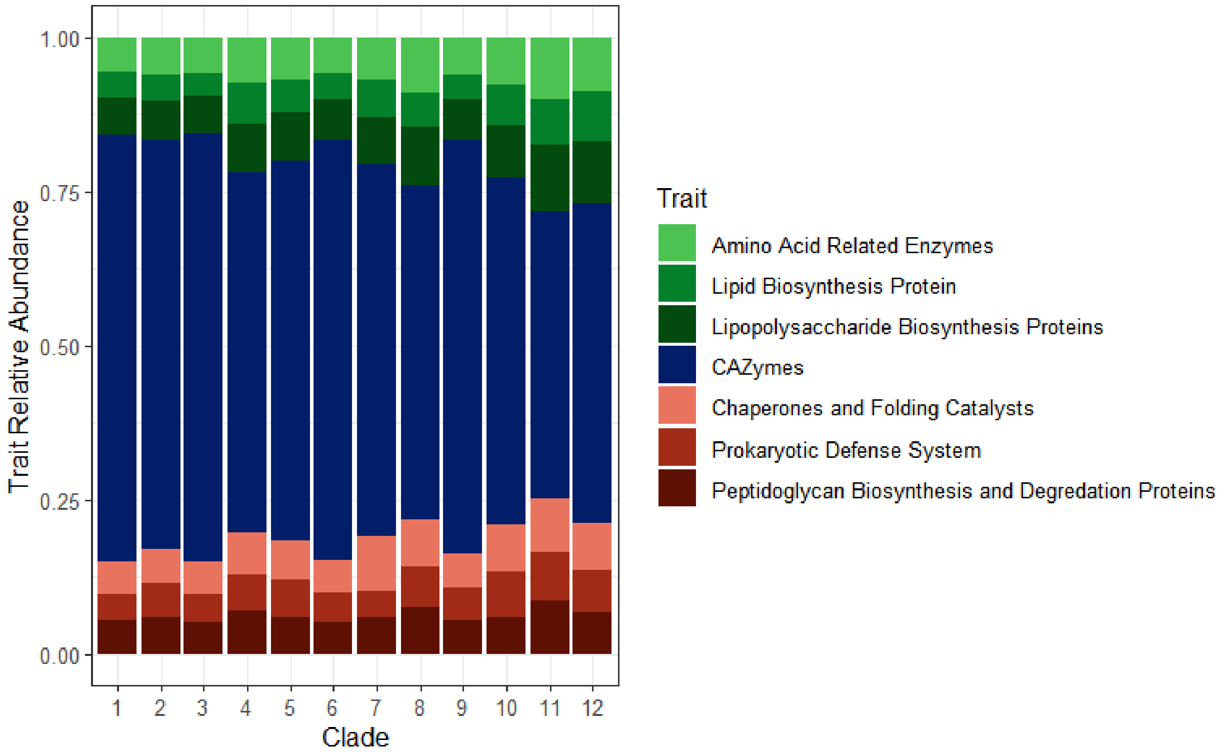

Supplemental Figure 4. Relative abundances of *Sphingomonas* habitat preference traits organized by clades. Green traits reflect the growth life history strategy, the blue CAZymes represent resource acquisition, and the red traits aid in stress tolerance.

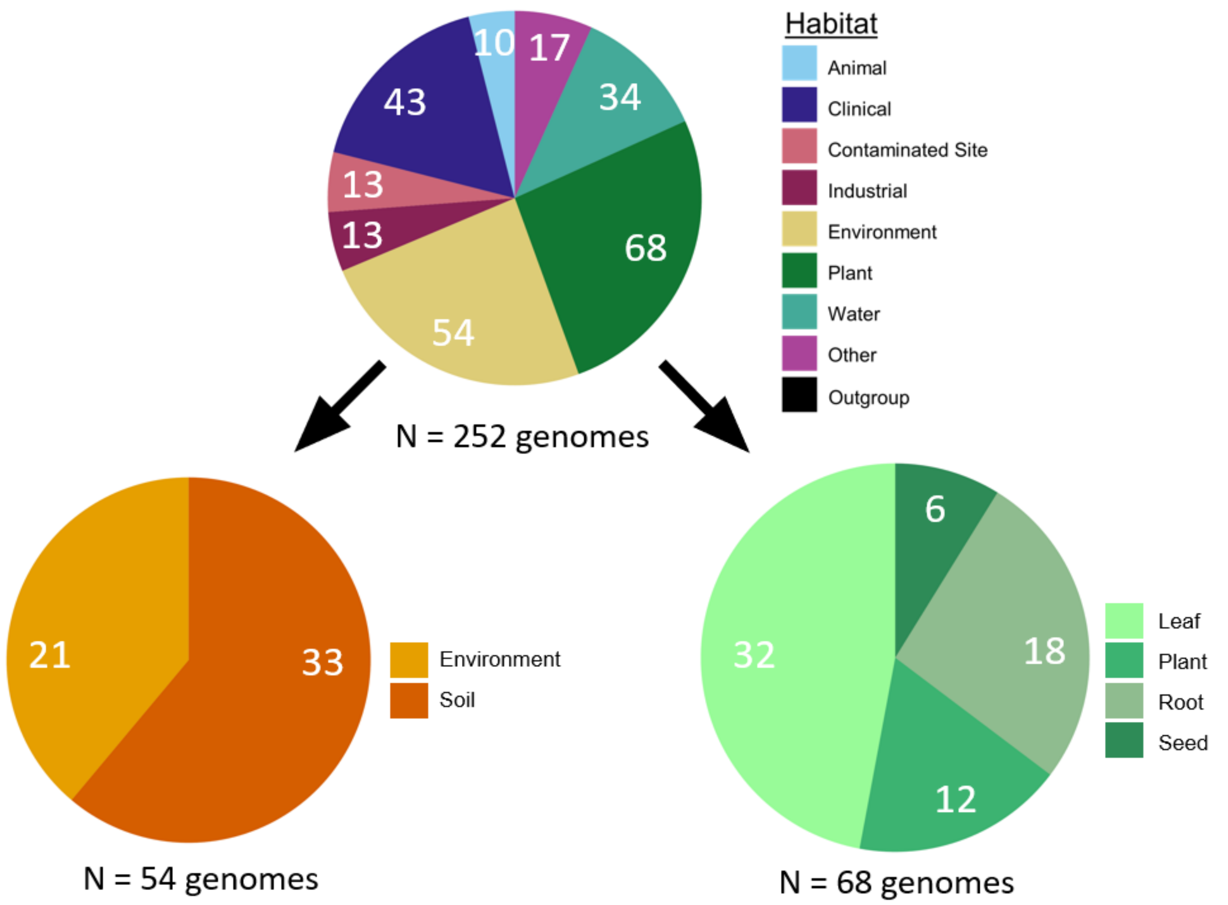

Supplemental Figure 5. Demographic information of *Sphingomonas* genomes (N = 252) where the environment (N = 67) and plant (N = 60) classifications are further broken down into additional subcategories.
